# Empowering women: overcoming barriers to breast cancer screening in Pakistan

**DOI:** 10.1097/MS9.0000000000004228

**Published:** 2025-12-11

**Authors:** Sidhant Ochani, Bilquees Fatima, Khushi Ochani, Sumran Azam, Kaleem Ullah, Md. Asaduzzaman Nur

**Affiliations:** a Department of Medicine, Khairpur Medical College, Khairpur Mir’s, Pakistan; b Department of Medicine, Islam Medical College, Sialkot, Pakistan; c Department of Dentistry, Dow University of Health Sciences, Karachi, Pakistan; d Department of Medicine, Karachi Medical and Dental College, Karachi, Pakistan; e Department of Hepatobiliary Surgery, Al-Amiri Hospital, Kuwait City, Kuwait; f Faculty of HPB Surgery, Enam Medical College, Dhaka, Bangladesh

**Keywords:** awareness campaigns, breast cancer, early detection, Pakistan, sociocultural barriers

## Abstract

The burden of breast cancer in Pakistan is staggering, with high incidence and mortality rates exacerbated by limited healthcare infrastructure, societal taboos, and cultural barriers. This manuscript reviews the challenges facing breast cancer detection and treatment in Pakistan, including late-stage diagnoses, inadequate access to medical facilities, and sociocultural norms inhibiting open dialogue and early intervention. Despite government initiatives and nongovernmental organization efforts, awareness remains low, with many women resorting to traditional healers or delaying medical attention due to financial constraints and cultural stigma. The manuscript highlights the critical need for comprehensive awareness campaigns targeting both urban and rural populations, supported by mobile clinics and mass media dissemination. It advocates for training healthcare workers, subsidizing screening methods, and engaging communities and international partners to improve access and affordability. By implementing these recommendations, Pakistan can make significant strides in early detection and treatment, ultimately alleviating the burden of breast cancer on its population and improving survival rates.

## Introduction

Breast cancer is the leading cancer in women and the second leading cause of cancer mortality. In Pakistan, it is estimated that more than 83 000 cases of breast cancer are reported each year. Every year, about 40 000 women die as a result of this devastating condition[1]. Pakistani women show an incidence rate of 50.1/100 000. India neighboring Pakistan, having a similar sociocultural background, similar breast-feeding practice, and first childbirth at a young age, has an incidence rate of only 19/10 000. Also, the mortality due to breast cancer in Pakistan is highest among all Asian countries[1]. This manuscript aims to review the critical challenges impeding early breast cancer detection and effective treatment in Pakistan, including late-stage diagnoses, inadequate access to facilities, and significant sociocultural barriers. It further advocates for a set of evidence-based recommendations to improve screening and treatment outcomes, ultimately alleviating the burden of breast cancer on the population. This study has been reported in line with the TITAN guidelines 2025[2].

## Sociocultural barriers

Pakistan is a developing country, and most two-third of the population lives in rural areas. There is a lack of basic health infrastructure in most rural areas of the country. Illiteracy is also a major factor in the lack of health awareness regarding various cancers including breast cancer among women. Thousands of women die each year due to a lack of awareness, as many of them ignore their health issues. The prognosis of breast cancer in the Pakistani population is poor as almost 70% of patients with breast cancer present to clinics with advanced disease, i.e., Stage III and IV. Most of them do not seek treatment due to a lack of awareness, societal taboos, and stigmas attached to the disease. Most Pakistani women have no proper access to medical facilities due to gender-based discrimination. At some stage of life, one in nine Pakistani women will become patients with breast cancer[3].

## Cultural stigma and privacy

In Pakistan, cultural norms dictate that discussions about breast health are considered private and often tied to matters of sexuality. This prevailing silence creates a barrier to open dialogues, leading to a lack of awareness regarding potential breast health issues. Consequently, information on breast cancer screening remains scarce, leaving many women unfamiliar with self-examinations, further impeding their ability to recognize abnormal lumps. Additionally, breast cancer carries a significant cultural taboo, profoundly affecting body image and perceptions of femininity. Those affected often associate the disease with diminished self-worth and discomfort, as it directly impacts a symbol of womanhood.

## Modesty and gender-based discrimination

These cultural beliefs also influence healthcare decisions, with many women opting to avoid male doctors due to considerations of modesty and religious beliefs. This can result in delayed medical attention, as they fear the social consequences of exposing their bodies to anyone other than their spouses. Ultimately, the collective impact of these cultural norms leads many women to postpone necessary treatment, convinced that such discussions and examinations conflict with deeply ingrained notions of modesty and piety[3].

## Economic hardship and alternative treatments

Understaffed public hospitals struggle with limited capacity, forcing them to reject many cancer patients. This also hinders early detection, as most health centers lack essential screening resources. Even equipped facilities often fail to meet patient needs, posing a significant challenge to providing sufficient care for cancer patients in the region, further delaying intervention and treatment. Insufficient awareness regarding breast cancer, lower socioeconomic status, and limited access to advanced medical facilities contribute to the delay in diagnosing breast cancer in Pakistani women, often a decade later than in Western women[4].

Additionally, the prevailing patriarchal societal structure constrains economic opportunities for women, rendering them financially dependent. Empirical studies from Pakistan emphasize the substantial barrier posed by economic hardships to breast cancer screening and treatment^[5,6]^. In response to these challenges, individuals often resort to alternative avenues, such as seeking solace in religious practices or turning to traditional healers instead of conventional hospital care. In traditional societies, spiritual interventions are often sought initially, deferring clinical intervention until the tumor progresses[7]. A study found that 40.7% of Pakistani women utilized alternative medicines, 17.1% ignored painless lumps, and 10.6% delayed seeking medical attention due to viewing the breast as a private organ[5].

## Comprehensive awareness campaigns

In Pakistan, healthcare focuses on patients, medicines, and customers, neglecting to promote disclosure of progressing disorders. This is due to low routine check-ups, high consultation fees, and limited counseling time. Recently, public health, cancer hospitals, and institutes launched a breast cancer awareness program with support from nongovernmental organizations (NGOs). Its goal is to educate about consequences, symptoms, risks, diagnosis, and treatments. The program uses electronic/print media and seminars on World Breast Cancer Day. However, only a few hospitals offer early detection services. These efforts, known as “Breast Clinic Day,” operate under PAEC-affiliated hospitals. Through this, women learn self-inspection methods for early detection. The health departments of the Government of Pakistan have valuable personnel resources, like nurses, female medical officers, and health visitors. Deploying and training them in rural health centers, basic units, and hospitals could be transformative in breast cancer diagnosis. This approach can significantly enhance survival rates and decrease mortality[6].

Pakistan’s government has taken initiatives to start an awareness campaign via SMS, recorded messages over phones, and displaying the color pink on public and private buildings. Pink Ribbon Pakistan is a non-funded, self-sustaining organization in Pakistan that has been dedicated to working on the issue of breast cancer with nationwide outreach since 2004. They are creating widespread awareness through community engagement on prevention, early detection, and increased access to treatment, including patient-aid and service delivery. Pink Tea Parties (PTP) are another fun way encouraged by Shaukat Khanum Hospital by providing free PTP kits that contain informative handouts about breast cancer awareness, which comforts women to raise awareness and hope in their circles and motivates them to build a better future for their children[8].

## Evidence-based recommendations

This disease often manifests without early warning signs, making it challenging to detect in its initial stages. Inadequate health education and limited access to regular check-ups further hinder timely identification. Consequently, malignant cases are frequently diagnosed when they have progressed significantly. Detecting the disease early, however, significantly enhances the likelihood of successful treatment. Hence, there is a crucial need to emphasize early detection measures for improved prognosis and outcomes[9]. Detecting breast cancer in its early stages is pivotal for a patient’s chances of survival and her overall quality of life during treatment and beyond. A study conducted in China involving 267,040 women who performed breast self-examination (BSE) demonstrated that, after approximately ten years of follow-up, BSE facilitated the early detection of cancers[10]. Similarly, a study conducted in Canada involving 290 000 women indicated that a screening protocol comprising a clinical examination and a mammogram achieved a remarkable 95% effectiveness in detecting breast cancer[11].

First and foremost, awareness campaigns must be launched nationwide, reaching both urban and rural populations. Mobile screening units (MSUs) should be deployed to remote and underserved areas, offering mammograms and clinical breast exams. Studies show that MSUs primarily reach women who would otherwise not be screened at fixed sites. For example, a study found that the mobile mammography population had a significantly higher percentage of uninsured patients (39%) compared to a stationary center (4%). Mobile units are an additive, rather than substitute, approach to screening, successfully reaching rural, low-income, and minority communities.^[12,13]^ MSUs can overcome some of the barriers, save money and time, and this is particularly important for asymptomatic women who cannot afford to lose their daily wages to travel long-distances to the clinic settings. We are reporting the findings of our cervical cancer screening initiative using the MSU.[14] In Pune, India, an MSU was used successfully for cervical cancer screening, demonstrating that mobile units can provide services “practically at the door step” for women in densely populated urban and rural areas, overcoming barriers of cost and travel time.[14] The effectiveness of this approach is supported by strong evidence, particularly in low-resource settings. Mobile clinics are proven to increase screening adherence among women who face structural barriers like lack of transportation or insurance, with studies showing they increase participation rates by a median of nearly 18 percentage points. Furthermore, similar multi-purpose mobile units are already being deployed in the region, including in Pakistan, to address the lack of health services in rural and remote communities.^[12,15]^

Leverage mass media, including television, radio, and social media, to disseminate information and success stories about breast cancer screening. Additionally, healthcare workers, including midwives and nurses, should receive training to conduct breast exams and educate women about self-breast examinations. Financial support and subsidies should be made available for mammograms and other screening methods, particularly for low-income women. Community engagement and support are essential, involving local leaders and NGOs in awareness and screening programs. Health insurance policies covering breast cancer screening should be advocated to make these services more affordable. Research on state health insurance mandates requiring coverage of screening mammograms found that these mandates significantly increased mammography screenings by 4.5–25%. The effects were larger in states that banned deductibles, suggesting that removing out-of-pocket costs is a key factor in increasing utilization.[16] Collaborating with international organizations and staying updated on screening technologies and treatment advancements can further bolster these recommendations. By implementing these measures, Pakistan can make substantial progress in improving breast cancer screening rates and ultimately reducing the burden of this disease on its population.

## Data Availability

Available upon reasonable request from the corresponding author.

## References

[1] Sohail S Alam SN. Breast cancer in Pakistan - awareness and early detection. Journal of the College of Physicians and Surgeons Pakistan JCPSP 2007;17:711–12. 18182132

[2] Premier Science, London, UK. Agha R Mathew G Rashid R. Transparency in the reporting of Artificial INtelligence – the TITAN guideline. Premium Journal of Science. 2025;10, p.100082

[3] Menhas R Umer S. Breast cancer among Pakistani women. Iran J Public Health 2015;44:586–87. 26056679 PMC4441973

[4] Saeed S Asim M Sohail MM. Fears and barriers: problems in breast cancer diagnosis and treatment in Pakistan. BMC Womens Health 2021;21:151. 33853583 10.1186/s12905-021-01293-6 PMC8045297

[5] Khan MA Hanif S Iqbal S. Presentation delay in breast cancer patients and its association with sociodemographic factors in North Pakistan. Chinese Journal of Cancer Research Chung-Kuo Yen Cheng Yen Chiu 2015;27:288–93. 26157325 10.3978/j.issn.1000-9604.2015.04.11 PMC4490192

[6] Gulzar F Akhtar MS Sadiq R. Identifying the reasons for delayed presentation of Pakistani breast cancer patients at a tertiary care hospital. Cancer Manag Res 2019;11:1087–96. 30774437 10.2147/CMAR.S180388 PMC6357878

[7] Brawley OW. Risk-based mammography screening: an effort to maximize the benefits and minimize the harms. Ann Intern Med 2012;156:662–63. 22547477 10.7326/0003-4819-156-9-201205010-00012

[8] BCA. Shaukat Khanum Memorial Cancer Hospital and Research Centres - official website. Accessed October 16, 2025. https://shaukatkhanum.org.pk/bca/

[9] Milosevic M Jankovic D Milenkovic A. Early diagnosis and detection of breast cancer. Technol Health Care 2018;26:729–59. 30124455 10.3233/THC-181277

[10] Thomas DB Gao DL Ray RM. Randomized trial of breast self-examination in Shanghai: final results. J Natl Cancer Inst 2002;94:1445–57. 12359854 10.1093/jnci/94.19.1445

[11] CBC. Value of clinical breast screening uncertain. CBC News. https://www.cbc.ca/news/science/value-of-clinical-breast-screening-uncertain-1.820325. September 1, 2009. Accessed 16 October 2025.

[12] New study shows mobile mammography reach is highest in underserved groups with low breast cancer screening adherence. Harvey L. Neiman Health Policy Institute. December 13, 2024. Accessed 16 October 2025. https://www.neimanhpi.org/press-releases/new-study-shows-mobile-mammography-reach-is-highest-in-underserved-groups-with-low-breast-cancer-screening-adherence/

[13] Stanley E Lewis MC Irshad A. Effectiveness of a mobile mammography program. Am J Roentgenol 2017;209:1426–29. 28871806 10.2214/AJR.16.17670

[14] Joshi S Muwonge R Kulkarni V. Mobile Screening Unit (MSU) for the implementation of the “screen and treat” programme for cervical cancer prevention in Pune, India. Asian Pacific Journal of Cancer Prevention APJCP 2021;22:413–18. 33639655 10.31557/APJCP.2021.22.2.413 PMC8190336

[15] 6. Mobile Medical Units (MMU) in rural areas in seven countries. Accessed 16 October 2025. https://www.isdb.org/kaap/projects/6-mobile-medical-units-mmu-in-rural-areas-in-seven-countries

[16] Bitler MP Carpenter CS. Health insurance mandates, mammography, and breast cancer diagnoses. American Economic Journal: Economic Policy 2016;8:39–68. doi:10.1257/pol.20120298. 29527253 PMC5844506

